# Non operative management of abdominal trauma – a 10 years review

**DOI:** 10.1186/1749-7922-8-14

**Published:** 2013-04-05

**Authors:** Mohsin Raza, Yasser Abbas, Vanitha Devi, Kumarapuram Venkatachalam Souriarajan Prasad, Kameel Narouz Rizk, Permasavaran Padmanathan Nair

**Affiliations:** 1 Surgery Department, Khoula Hospital, Muscat, Sultanate of Oman; 2 4/894, AikMinar Enclave, Near ShaukatManzil, Dodhpur, Aligarh, UP 202002, India

**Keywords:** Non-operative management, Advanced Trauma life Support, Surgery

## Abstract

**Introduction:**

Due to high rate of operative mortality and morbidity non-operative management of blunt liver and spleen trauma was widely accepted in stable pediatric patients, but the general surgeons were skeptical to adopt it for adults. The current study is analysis of so far largest sample (1071) of hemodynamically stable blunt liver, spleen, kidney and pancreatic trauma patients managed non operatively irrespective of severity of a single /multiple solid organ injury or other associated injuries with high rate of success.

**Methods:**

Experience of 1071 blunt abdominal trauma patients treated by NOM at a tertiary care National Trauma Centre in Oman (from Jan 2001 to Dec 2011) was reviewed, analyzed to determine the indications, methods and results of NOM. Hemodynamic stability along with ultra sound, CT scan and repeated clinical examination were the sheet anchors of NOM. The patients were grouped as (1) managed by NOM successfully, (2) failure of NOM and (3) directly subjected to surgery.

**Results:**

During the 10 year period, 5400 polytrauma patients were evaluated for abdominal trauma of which 1285 had abdominal injuries, the largest sample study till date. Based on initial findings 1071 patients were admitted for NOM. Out of 1071 patients initially selected 963 (89.91%) were managed non operatively, the remaining 108 (10.08%) were subjected to laparotomy due to failure of NOM. Laparotomy was performed on 214(19.98%) patients as they were unstable on admission or had evidence of hollow viscous injury.

**Conclusion:**

NOM for blunt abdominal injuries was found to be highly successful in 89.98% of the patients in our study. Management depended on clinical and hemodynamic stability of the patient. A patient under NOM should be admitted to intensive care / high dependency for at least 48-72 hours for close monitoring of vital signs, repeated clinical examinations and follow up investigations as indicated.

## Introduction

Nearly six thousand men, women and children have lost their lives in road traffic crashes in Oman between 2000 and 2008. Seventy thousand injured and many disabled for life (Survey by German Institute of Technology in Oman).

Abdominal injuries occur in 31% patients of polytrauma with 13 and 16% spleen and liver injuries respectively, and pelvic injuries in 28% of cases, making differential diagnosis between pelvic or intractable abdominal injury difficult [1,2].The haemodynamically unstable patients with frank signs of exsanguination have to undergo laparotomy, however, selecting these patients, especially in the polytrauma remains a challenge.

High rate of operative complications caused paradigm shift from operative to non-operative management (NOM) in hemodynamically stable blunt abdominal trauma patients [3,4]. NOM can be safely practiced in a Trauma Care Centre which has Trauma Surgeons, newer imaging modalities, High Dependency Unit (HDU), ICU and other supporting services [5]. Repeated clinical examination supplemented with modern imaging and laboratory investigations play a key role in reaching therapeutic decisions, thus preventing unnecessary laparotomies. Liver being a sturdy organ has a higher success NOM rate, exceeding 90% [6,7]. Haemodynamically stable liver and spleen injuries can be managed conservatively irrespective of the grade of injury [8-10]. NOM is also highly successful in case of renal trauma with success rates over 90% [11].

NOM of solid abdomen organ injuries is now established for hemodynamically stable patients. The present study is retrospective analysis and outcome of operative and NOM of blunt abdominal injuries in polytrauma at a Tertiary Care trauma Centre. Hemodynamically unstable patients with frank signs of exsanguination underwent urgent laparotomy, however, decision in polytrauma remains a challenge [12].

## Material and methods

This is a ten year (January 2001 to December 2011) retrospective analysis of successful implementation of NOM for blunt abdominal trauma at a Tertiary Trauma Care Center in Oman. Oman has one of the highest incidences of Road traffic accidents in the world. Almost all the patients were victims of road traffic accidents. Being National trauma center, our hospital receives patients from all primary and secondary care hospitals in Oman, in addition to direct admission through accident and emergency.

On arrival all the patients were assessed and resuscitated if necessary, in accordance with ATLS protocol. History including the mechanism of injury formed an important part of the evaluation. All the patients underwent FAST/Abdominal sonography. Stable patients with positive FAST were further evaluated with chest, abdomen and pelvic CT scan. Patients with other associated injuries were examined by the respective specialists with close coordination. Patients with heart rate of <110/min, systolic BP of >90 mm Hg on arrival or following initial resuscitation were considered stable. Prior to the inclusion of the patients in the study an ethical clearance was sought from the competent authority of the Khoula Hospital, Oman. Written informed consent was obtained from the patient/close relatives for publication of this report and any accompanying images.

Among 5400 polytrauma patients, 1285 were diagnosed to have abdominal injuries. On secondary survey, based on hemodynamic stability, clinical findings and investigations, 1071(83%) patients were selected for NOM. The exclusion criteria for rejecting NOM in 214(17%) patients were signs of exsanguination, persistent hemodynamic instability and no response to initial resuscitation or obvious bowel injury. All stable patients were treated nonoperatively. The severity of head injury, associated orthopedic injuries, a high injury severity score or a higher radiological grading of the visceral injuries or multiple solid organ trauma were not considered as an exclusion criteria in haemodynamically stable patients.

NOM patients were admitted to HDU/ICU, closely monitored with repeated clinical assessment. The protocol included evaluation of vitals, Pulse, BP, temperature, urine output, 12 hourly hemoglobin, and hematocrit (HCT) estimation for the first 72 hrs. Follow up ultra sound abdomen or CT scan were done only if hemoglobin dropped despite 3 units of blood transfusion, progressive distension of abdomen, signs of infection, vomiting, hematuria or tachypnea. To detect occult bowel injuries, not able to diagnose otherwise, diagnostic peritoneal tap was notably successful.

NOM was successful in 963(89.91%) out of 1071 patients. Whereas, 108 patients showed signs of ongoing hemorrhage, delayed evidence of hollow viscous perforation, or intra-abdominal infection requiring laparotomy. They were grouped in NOM failed category.

### Statistical analysis

The percent differences were calculated between the operated and nonoperated groups. Student’s ‘t’ test was used for statistical analysis, p values < 0.05 were considered to be statistically significant.

## Results

A total of 5400 patients were evaluated for abdominal trauma during ten year period from January 2001 to December 2011. Various types of blunt abdominal injuries were found in 1285 patients. After initial evaluation, non-responders to resuscitation, 214 hemodynamically unstable patients were operated, while, 1071 patients were initially selected for NOM, but NOM failed in 108 patients.

Males dominated in both groups with no significant difference in age, co-morbidities, and mechanism of injury (Table 1). Operated group presented with low systolic BP (<90 mm Hg), tachycardia, low haematocrit and higher blood transfusion requirement (Table 1). Intubation was done in 95% of patients in the Emergency Department.

**Table 1:** Comparison of various parameters in NOM-S, NOM-F and Operative groups and demographic, admission and injury characteristics

	**NOM-S group**	**NOM-F group**	**Operative- group**
	**n = 963**	**n = 108**	**n = 214**
Age	25.31#	35.21#	31.26*#
Male sex	558(58%)	73(68%)	132(62%)
RTA	895(93%)	99(92%)	201(93%)
ISS	37.09# ±1.58	41# ±2.25	40.93*# ±2.25
Haematocrit on admission	36.62# ±3.97	31.83# ±2.67	27.53*# ±2.89
SBP > 90mmhg	885(92%)	68(63%)	25(12%)
Heart rate < 110/min	799(83%)	92(85%)	203(95%)
Blood transfusion	2.77# ±0.85	5.10# ± 0.96	5.57*# ±0.87
Positive FAST	818(85%)	102(94.4%)	214(100%)
Co- morbidities	404(42%)	96(45%)	71(66%)
Liver Injury	320(33%)	0	29*(13.55%) ±1.64
Splenic injury	288(30%)	16(15%)	37*(17.3%) ±0.35
Others	355(37%)	92(85%)	148*(69.16%) ±1.92

RTA Road Traffic Accident, ISS Injury Severity Score, SBP Systolic Blood Pressure, FAST Focused Abdominal Sonography for Trauma.

Values are #Mean ± SEM. The *p < 0.05 were considered as significant as compared to NOM-S and Operative groups.

**Figure 1:**
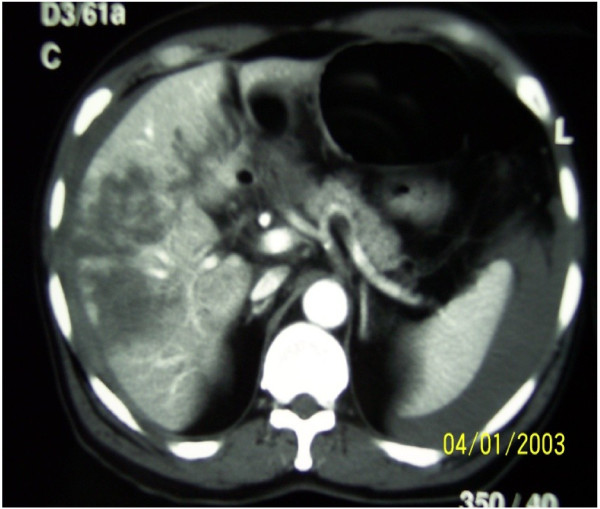
The picture shows severely injured liver.

**Figure 2:**
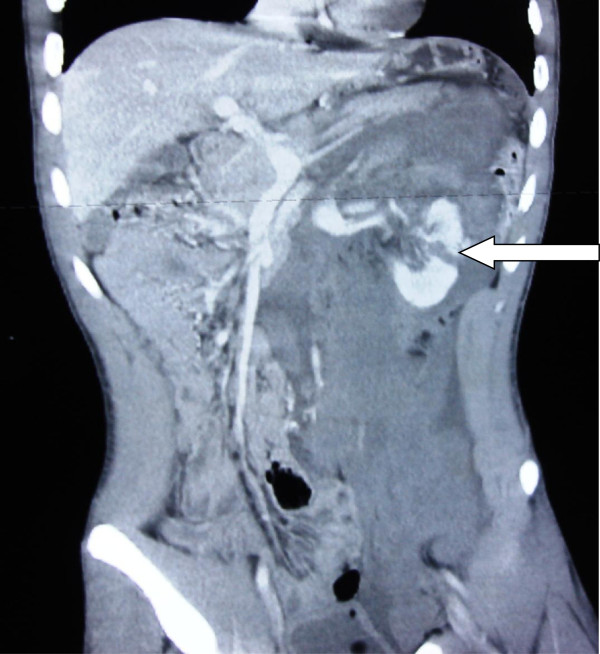
Severe renal injury with a midline shift, successfully managed non operatively, arrow showing injured kidney.

**Figure 3:**
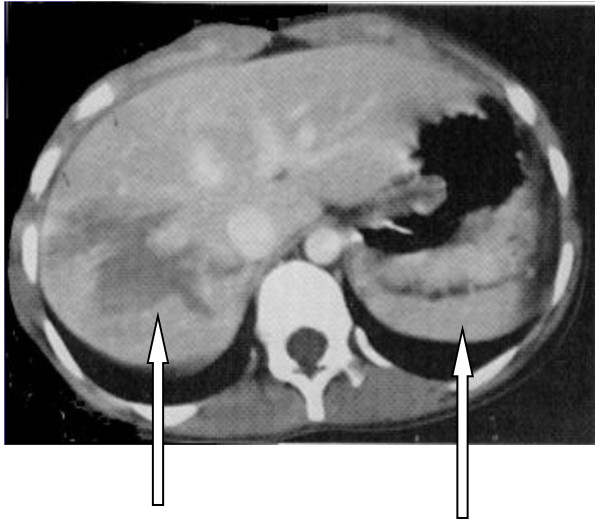
Shows both liver and splenic injuries indicated by arrows.

**Figure 4:**
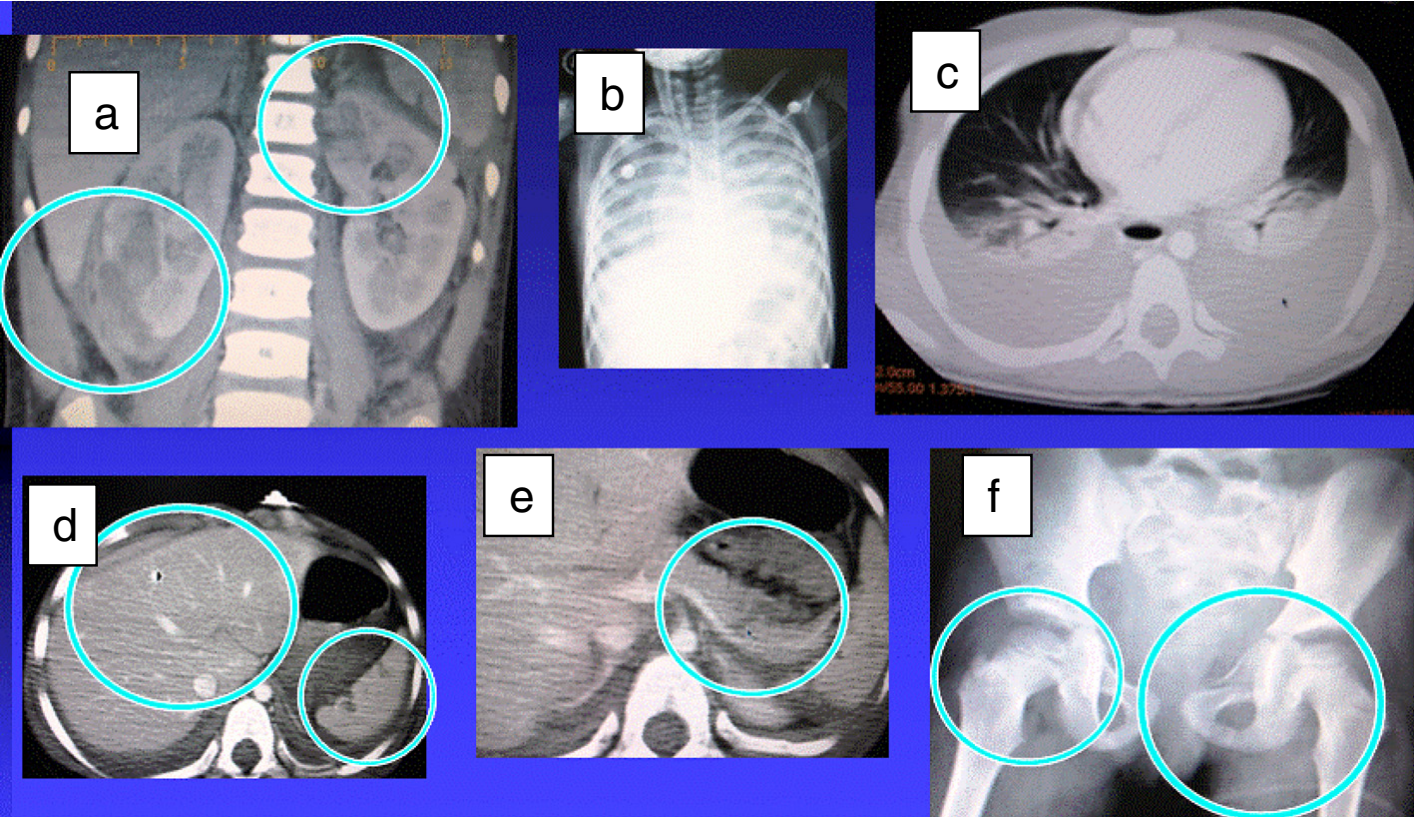
Shows all the solid organ injuries with bilateral haemothorax and fractures: A girl aged 6 years had injuries in all the solid organs (a) both kidneys,(b) and (c) bilateral haemothorax (d) liver and spleen, (e) body of pancreas, (f) bilateral acetabular fractures were treated non operatively except bilateral intercostal drains were inserted.

Most of the patients had polytrauma, hence no significant difference in the Injury Severity Score (ISS) was appreciated between the two groups (Table 1). FAST was positive in 100% in the operated group. No significant difference was noted between the NOM and the operated group in relation to the liver, spleen and multiple abdominal injuries (Table 1). NOM failure group had multiple solid organ injuries in 92(85%) patients. We could easily manage the patients with severe isolated liver (Figure 1), spleen and kidney injuries (Figure 2). Both liver and spleen were injured in 15.6% patients (Figure 3), while 21 patients (1.9%) had three solid organs liver, spleen and kidney injured. One 6 year old girl had liver, spleen, pancreas, bilateral kidney injuries with bilateral hemothorax and bilateral pelvic acetabular fracture, was successfully managed non-operatively (Figure 4), 196 (18.3%) patients had multiple organ injury associated with retroperitoneal hematoma and fractures (Table 2).

**Table 2:** Distribution of NOM patients according to their organ injury

**Organs injured in nom patients**	**Number**	**Percentage**
Liver Injury Isolated	320	29.8
Spleen Isolated Injury	304	28.3
Kidney Isolated Injury	052	05.2
Pancreatic injury	4	0.3
Ureteric Injury	3	0.2
Urinary Bladder (Intraperitoneal)	1	0.09
Liver/Spleen	168	15.6
Liver/Spleen/Kidney	21	1.9
Liver/Spleen/Kidney/Pancreas	1	0.09
Bilateral Kidney Injury	1	0.09
Others (Multiple organ injuries with associated retroperitoneal haematoma with pelvic fractures)	196	18.3

The operated group had an ICU admission rate of 57%, with a longer period of hospitalization (23.31 days) and higher morbidity (16%) in comparison to the NOM with an ICU admission rate of 24%, length of stay (10.23 days) and morbidity of (<1%) (Table 1). In the operative group six patients died.

In the NOM failure group 16 patients had delayed splenic bleed presenting between 24 hours and 10 days. Delayed small bowel rupture was observed in 21 patients. Bowel injury was missed on the initial CT scan in 3 patients. Ongoing mesenteric vessel bleed with delayed bowel ischemia occurred in 37 patients. Intraperitoneal urinary bladder tear was missed in 5 cases, non-therapeutic laparatomies done in 28 cases of retroperitoneal hematoma. Sigmoid colon injury diagnosis was masked and delayed for 24 hours due to severe head injury associated with fracture femur in one patient, causing mortality.

**Figure 5:**
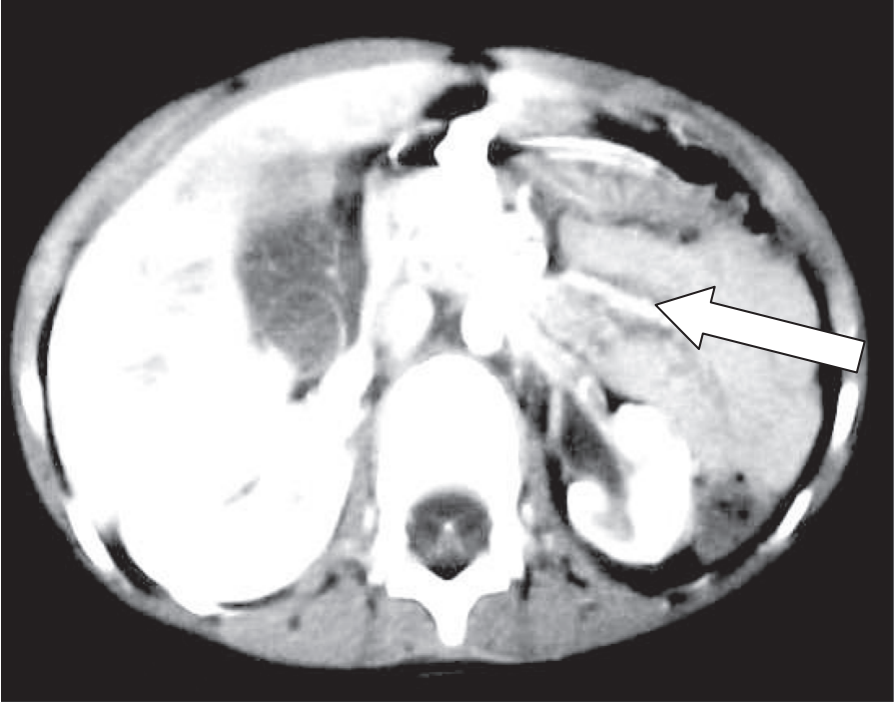
Subserous extravasation of dye causing a fuzzy mesentry is suspicious of mesenteric vascular disruption.

**Figure 6:**
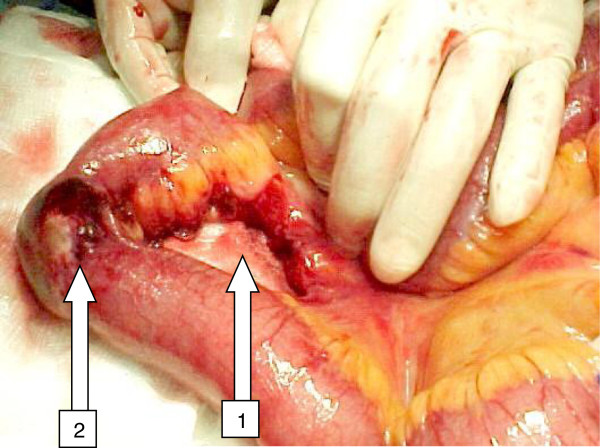
**Mesentric vascular injury showing bowel wall necrosis and delayed perforation: Mesenteric injury (1) caused bowel ischemia but bowel wall necrosis and perforation occurred late on third day (2).** Such patients have an unexplained high pulse rate.

Sub serous extravasations of dye in contrast CT (Figure 5), bowel wall thickening or mesenteric fat streaking may not be very reliable signs but suspicious of mesenteric injury. It causes ischemia but may take 2-3 days to cause perforation. We observed an unexplained tachycardia, while the ischemic process in the bowel goes on. Patients kept passing stools for 3-4 days after trauma until the ischemic bowel wall ruptured causing peritonitis (Figure 6).

## Discussion

Sir McCormack in 1900 was the first to advocate “A man wounded in war in the abdomen dies if he is operated upon and remains alive if he is left in peace” [13]. This aphorism was a surgical doctrine to manage abdominal trauma in the warfield during early 20^th^ century. This practice went into oblivion due to dogma of mandatory laparotomy in every case of hemoperitonium.

The advent of newer imaging techniques with high resolution CT scanners has enabled the clinicians to exactly diagnose the extent of intra-abdominal organ injury [2]. With the publication of many reports of success during the last 20 years, NOM has become an established and accepted management protocol for solid organ injuries in hemodynamically stable patients [9,14].

NOM poses challenge to Trauma Surgeons on account of varied clinical picture on arrival. The associated injuries, alcohol and drugs may mask abdominal signs and symptoms. Patients with short pre-hospital transport time have initial subtle clinical features affecting early diagnosis. Around 20 to 40% patients with radiologically significant hemoperitoneum may not have any significant clinical findings. Hemodynamically stable patients with solid organ injury should be considered for NOM after ruling out bowel trauma. Published literatures and our study have shown that radiological grade of severity of injury is not a contraindication for NOM [15]. CT contrast blush from minor vessels in solid organs were managed by NOM with caution. However, a CT contrast blush of a major vessel in arterial / venous phase is indicative of ongoing hemorrhage, which portends NOM failure. Mesenteric injuries causing bowel ischemia remains a challenge [16]. Presence of fluid without solid organ injury is a significant marker of mesenteric or bowel injury [17]. Usefulness of CT in bowel injuries remains controversial [18].

Liver due to its firm texture is more confidently treated by NOM [19]. In our analysis NOM succeeded in all stable isolated liver injuries but failed in 15% isolated splenic trauma. Delayed splenic bleed occurred in 16(1.5%) of total 1071 patients with other associated injuries. Most splenic injuries did not require close observation beyond 3 days [14,20].

In x-ray, absence of free air under diaphragm or oral contrast leak does not rule out bowel injury. In suspected stable patients we have done peritoneal tap to look for bowel contents. In absence of perforation and to ensure cessation of intraperitoneal bleed and subsequent resorption of blood breakdown products from the vast peritoneal surface, we left the catheter in situ to drain out collected blood from peritoneal cavity, until it stops draining.

We have very good success rate in the management of high grade renal injuries conservatively and the same is recorded in other centers [11,21]. All extraperitoneal urinary bladder injuries were treated with transurethral catheter, including 4 patients with small intraperitoneal leaks.

Blood transfusion requirement, morbidity, mortality and incidence of non-therapeutic laparotomy were significantly reduced with NOM. The successful management depends on repeated clinical assessment preferably by the same clinical team in HDU/ICU, hemodynamic stability, serial determination of hemoglobin, haematocrit, WBC and follow up ultrasound/CT scan, if indicated. However, routine repeate CT scan is not essential in clinically improving patients. Thumping of chest for physiotherapy is strictly forbidden in splenic and liver injuries. Conscious patients not having spine, lower limb or pelvic fractures were mobilized within 48 hours. Initially hospital authorities and even our surgical colleagues were critical about NOM, but following successful results, NOM has now been accepted as a standard method of managing hemodynamically stable blunt abdominal trauma patients in most of the Trauma Centres including ours with a success rate of above 80% [4]. Heyn etal [12] suggested that in patients with multiple injuries abdominal ultra sound and CT have complementary value. Anatomical CT grading is an ineffective exclusion criterion for NOM or embolisation for splenic or hepatic trauma [15].

Earlier NOM was not preferred in polytraumatised patients but recently several reports of successful results in polytrauma with strict monitoring irrespective of age or other concomitant injuries have been reported [7,22] and the same is reproduced in our study.

Higher amount of blood transfusions were given to maintain hemodynamic stability in patients with associated long bone, pelvic fractures, retroperitoneal hematomas and hemothorax etc. Isolated liver, spleen or kidney injuries did not receive more than 3-4 pints of blood.

In our analysis we did not find any significant differences between the operated and NOM group in relation to the age, co- morbidities and mechanism of injury. But the operated group presented with poor hemodynamic stability thus necessitating increased blood transfusion and higher rate of intubation in the Emergency Department as compared to the NOM group.

As we look ahead the NOM will play major role in management of patients with blunt abdominal trauma.

## Conclusion

NOM for blunt abdominal trauma was found to be highly successful and safe in our analysis. Management by NOM depends on clinical and hemodynamic stability of the patient, after definitive indications for laparotomy are excluded. A patient under NOM should be admitted to ICU / HDU for at least 48-72 hours for close monitoring of vital signs and repeated clinical examinations. Follow up radiological investigations to be done as indicated. Higher anatomical image grading [3-5] of solid organ injury is not a deterrent to NOM. Even patients with multiple abdominal injuries can be successfully managed by NOM provided they are closely monitored. NOM has a significant decrease in lengt of hospital stay and morbidity compared to patients who undergo surgery. Fully equipped trauma care centres with available trauma surgeons willing to operate at any time is very important. NOM to be terminated if patient develops haemodynamic instability and appearance of new peritoneal signs due to delayed hollow viscous or missed injuries.

No procedure /practice are free from risk. Admission to ICU and its related problems, delay in diagnosis and management of missed bowel and vascular injuries are few of the risks involved in NOM. With newer modalities of imaging the percentage of delay in diagnosis is negligible.

## Abbreviations

HDU: High dependency unit; ICU: Intensive care unit; ATLS: Advanced life trauma support; FAST: Focused abdominal sonography in Trauma.

## Competing interests

The authors declare that they have no competing interests.

## Authors’ contributions

MR Head of the unit conceived the idea of the study, and also performed and supervised the whole process and operated when required, written and corresponded the manuscript. YA assisted in managing the patients with strict vigilance and helped in the preparation of manuscript. VD, KVSP, PPN assisted in managing the patients, performed and recorded repeated clinical assessments, acted upon and notified alarming changes in clinical features of the patients. KNR closely collaborated and supported the study, helped in preparation of manuscript discussed and critically analyzed the non operative management of patients in grand rounds on day to day basis. All authors read and approved the final manuscript.
